# Unusual manifestation of disseminated herpes simplex virus type 2 infection associated with pharyngotonsilitis, esophagitis, and hemophagocytic lymphohisitocytosis without genital involvement

**DOI:** 10.1186/s12879-019-3721-0

**Published:** 2019-01-17

**Authors:** Shuhei Kurosawa, Noritaka Sekiya, Kazuaki Fukushima, Kazuhiko Ikeuchi, Akito Fukuda, Hideyuki Takahashi, Fangyi Chen, Hideki Hasegawa, Harutaka Katano, Tsunekazu Hishima, Keigo Setoguchi

**Affiliations:** 1 Division of Hematology, Tokyo Metropolitan Cancer and Infectious Diseases Center Komagome Hospital, 3-18-22 Hon-komagome, Tokyo, 1138677 Bunkyo-ku Japan; 2 Department of Infection Prevention and Control, Department of Clinical Laboratory, Tokyo Metropolitan Cancer and Infectious Diseases Center Komagome Hospital, 3-18-22 Hon-komagome, Tokyo, 1138677 Bunkyo-ku Japan; 3 Department of Infectious Diseases, Tokyo Metropolitan Cancer and Infectious Diseases Center Komagome Hospital, 3-18-22 Hon-komagome, Tokyo, 1138677 Bunkyo-ku Japan; 4 Division of Rheumatology, Tokyo Metropolitan Cancer and Infectious Diseases Center Komagome Hospital, 3-18-22 Hon-komagome, Tokyo, 1138677 Bunkyo-ku Japan; 5 Department of Pathology, National Institute of Infectious Diseases, 1-23-1 Toyama, Tokyo, 1628640 Shinjyuku-ku Japan; 6 Department of Pathology, Tokyo Metropolitan Cancer and Infectious Diseases Center Komagome Hospital, 3-18-22 Hon-komagome, Tokyo, 1138677 Bunkyo-ku Japan

**Keywords:** Herpes simplex virus type 2, Disseminated infection, Hemophagocytic lymphohistiocytosis

## Abstract

**Background:**

Herpes simplex virus (HSV) has various presentations, depending on the patient’s immune status, age, and the route of transmission. In adults, HSV type 1 is found predominantly in the oral area, and HSV type 2 (HSV-2) is commonly found in the genital area. HSV-2 infection without genital lesions is uncommon. Herein we report a unique case of pharyngotonsillitis as an initial manifestation of disseminated HSV-2 infection without genital involvement.

**Case presentation:**

A 46-year-old male was admitted to our hospital with a 1-week history of fever and sore throat. His past medical history included hypereosinophilic syndrome diagnosed at age 45 years. Physical examination revealed throat congestion, bilaterally enlarged tonsils with exudates, tender cervical lymphadenopathy in the left posterior triangle, and mild epigastric tenderness. The laboratory data demonstrated bicytopenia, elevated liver enzyme levels, and hyperferritinemia. A bone marrow smear showed hypocellular marrow with histiocytes and hemophagocytosis. The diagnosis of HLH was confirmed, and the patient was treated with methylprednisolone pulse therapy on days 1–3. On day 5, despite initial improvement of the fever and sore throat, multiple, new, small bullae developed on the patient’s face, trunk, and extremities. Additional testing showed that he was positive for HSV-specific immunoglobulin M and immunoglobulin G. Disseminated HSV infection was suspected, and intravenous acyclovir (10 mg/kg every 8 h) was begun. A subsequent direct antigen test of a bulla sample was positive for HSV-2. Moreover, tonsillar and esophageal biopsies revealed viral inclusion bodies. Immunohistochemical staining and a quantitative real-time polymerase chain reaction (PCR) assay confirmed the presence of HSV-2. Disseminated HSV-2 infection with multiple bullae, tonsillitis, esophagitis, and suspected hepatic involvement was diagnosed. After a 2-week course of intravenous acyclovir, his hematological status and liver function normalized, and his cutaneous skin lesions resolved. He was discharged on day 22 in good general health and continued taking oral valacyclovir for viral suppression due to his immunosuppressed status.

**Conclusion:**

Disseminated HSV-2 infection should be considered as one of the differential diagnoses in patients with pharyngotonsillitis and impaired liver function of unknown etiology even if there are no genital lesions.

## Background

Herpes simplex virus (HSV) is a large, double-stranded DNA virus belonging to the ubiquitous *Herpesviridae* family of viruses, which includes the herpes simplex virus-1 (HSV-1), herpes simplex virus-2 (HSV-2), varicella zoster virus, cytomegalovirus, Epstein-Barr virus, human herpes viruses 6 and 7, and Kaposi’s sarcoma-associated herpesvirus (type 8) [1]. HSV has various presentations, depending on the immune status and age of the host and the route of transmission [2]. Although the typical clinical manifestations are gingivostomatitis and orolabial, ocular, and genital disease, this clinical picture may be complicated by disseminated infection in immunocompromised hosts and neonates [3].

HSV-1 infection usually involves the face and skin “above the waist.” On the other hand, HSV-2 infection usually involves the genitalia and skin “below the waist” in sexually active adolescents and adults [1]. Thus, HSV-2 infection “above the waist” without genital lesions is uncommon. Herein we report a unique case of disseminated HSV-2 infection complicated by hemophagocytic lymphohistiocytosis (HLH), which developed during immunosuppressive therapy for hypereosinophilic syndrome.

## Case presentation

A 46-year-old male was admitted to our hospital with a 1-week history of fever and sore throat. His past medical history included hypereosinophilic syndrome diagnosed at age 45 years. An extensive workup failed to disclose any underlying diseases. The patient had been receiving oral prednisolone (8 mg/day) and azathioprine (100 mg/day) regularly for approximately nine months, and his hematological status was stable. He denied any recent travel, illicit drug use or exposure to arthropods. He is married and reported monogamous sexual activity with his wife.

On initial presentation, his temperature was 39 °C, his blood pressure was 145/103 mmHg, his pulse rate was 111 beats per minute and regular, his respiratory rate was 20 breaths per minute, and his percutaneous oxygen saturation was 99% on ambient air. Physical examination revealed throat congestion, bilaterally enlarged tonsils with exudates, tender cervical lymphadenopathy in the left posterior triangle, and mild epigastric tenderness. An examination of the genital area revealed no abnormal findings such as an ulcer or blisters, and the remainder of the examination was unremarkable.

**Fig. 1:**
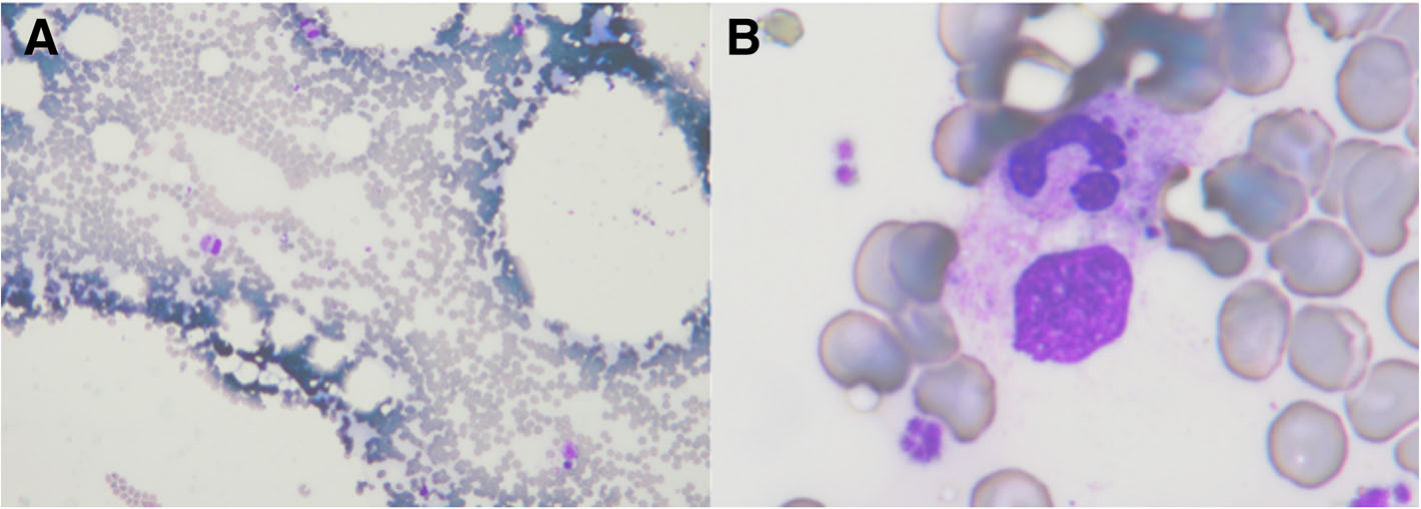
Bone marrow smear on admission showing hemophagocytosis with a Giemsa stain, which demonstrates many histiocytes with hemophagocytosis. **a**: Low power image. **b**: High power image

The laboratory data at admission demonstrated bicytopenia (white blood cell count: 1400 /μL; platelet count: 13.4 × 10^4^ /μL), elevated liver enzyme levels (aspartate aminotransferase (AST): 1558 U/L; alanine aminotransferase (ALT): 1007 U/L; lactate dehydrogenase (LDH): 2688 U/L; alkaline phosphatase: 265 U/L; total bilirubin: 0.9 mg/dL), and hyperferritinemia (11,480 ng/ml; normal range: 3.6–114.0 ng/ml). Serologic tests for hepatitis A, B, and C viruses and human immunodeficiency virus (HIV) were negative. Serum antibodies confirmed past infection by the Epstein-Barr virus, cytomegalovirus, and varicella zoster virus. Computed tomography demonstrated prominent hepatosplenomegaly and multiple low-density areas in the liver. An upper gastrointestinal endoscopy revealed multiple vitiligo lesions in the esophagus. A bone marrow smear showed hypocellular marrow with histiocytes (3.0%) and hemophagocytosis (Fig. 1). HLH was diagnosed based on the diagnostic criteria for HLH [4]. Given that the presenting symptom of pharyngotonsillitis is an initial manifestation, we suspected that the HLH was caused by an infectious etiology. There was no evidence of either bacterial or fungal infection in the blood and urine cultures or serological examinations. The patient received meropenem (1 g every 8 h) and intravenous methylprednisolone pulse therapy (1 g/day) on hospital days 1–3.

**Fig. 2:**
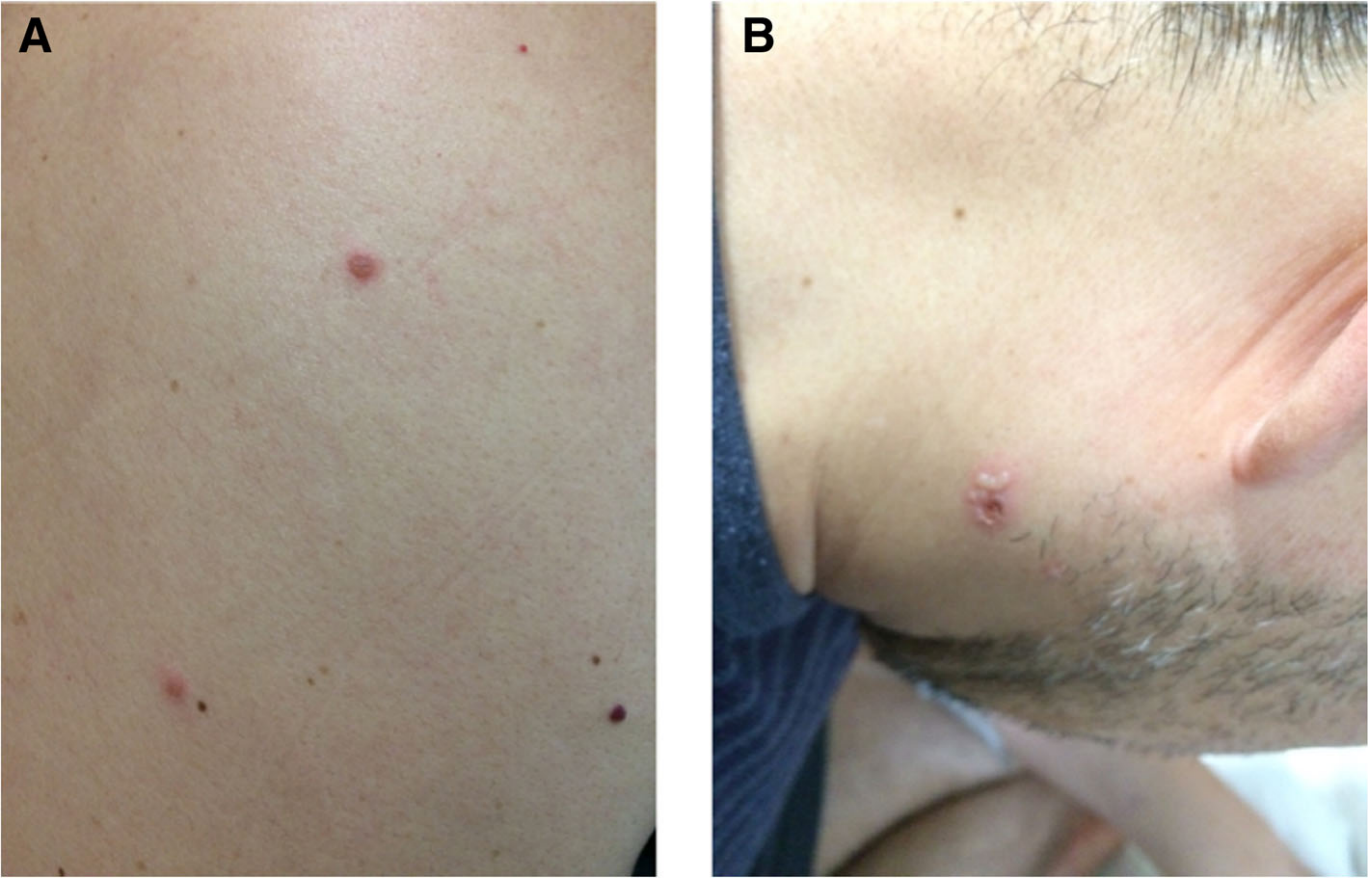
Multiple bullae 5 days after admission. **a**: trunk, **b**: face

**Fig. 3:**
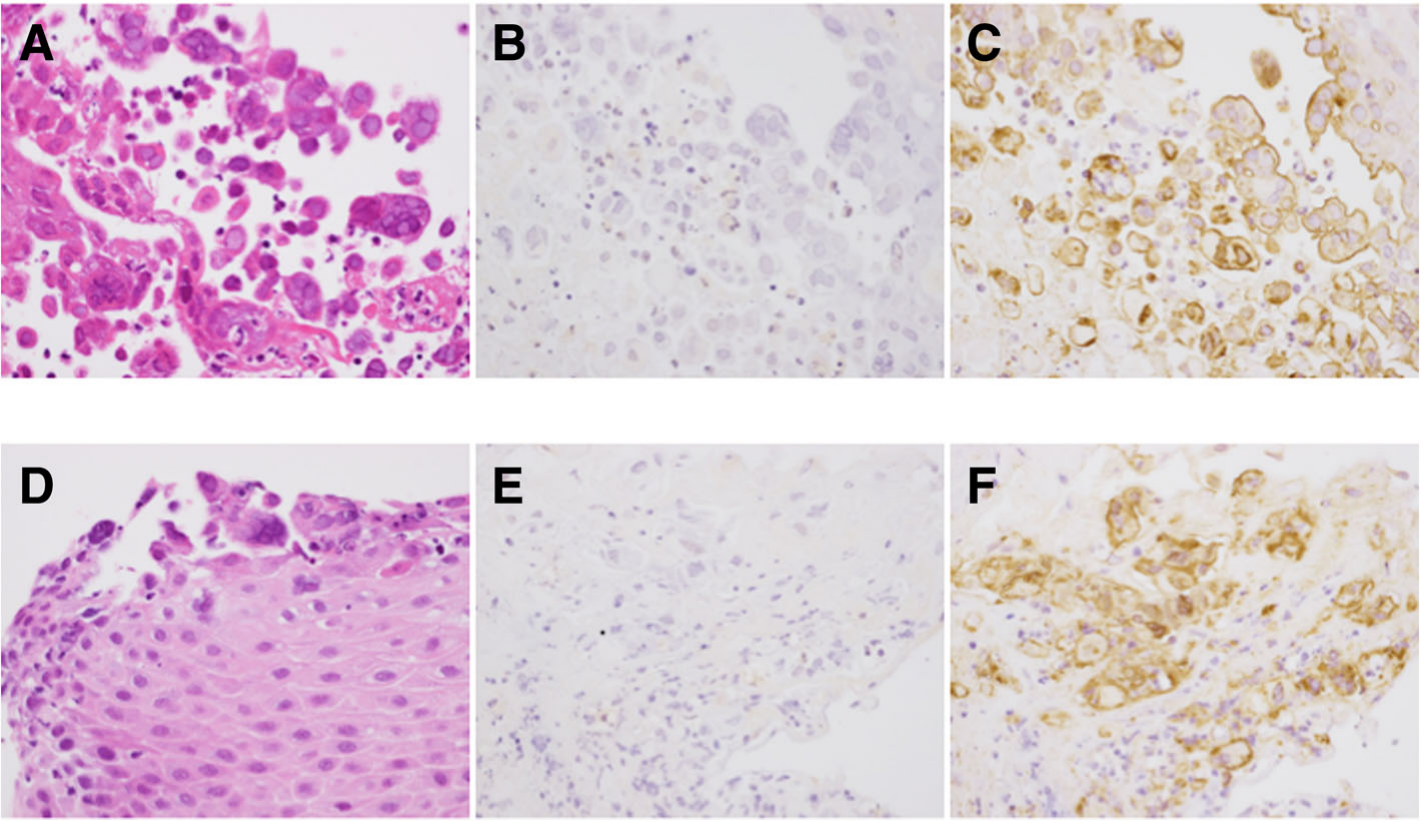
Histopathological findings of tonsillar (**a**-**c**) and esophageal (**d**-**f**) biopsies. **a**, **d**: Hematoxylin and eosin staining showing discohesive superficial squamous epithelial cells containing Cowdry type **a** or type **b** intranuclear inclusions. **b**, **e**: Negative immunohistochemical staining for HSV-1. **c**, **f**: Positive immunohistochemical staining for HSV-2

On day 5, despite initial improvement of the fever and sore throat, multiple, new, small bullae developed on the patient’s face, trunk, and extremities (Fig. 2), and the liver enzyme level remained elevated (AST: 935 U/L; ALT: 959 U/L; LDH: 2140 U/L). Additional testing showed positivity for HSV-specific immunoglobulin M (IgM) and immunoglobulin G. Disseminated HSV infection was suspected, and intravenous acyclovir (10 mg/kg every 8 h) and methylprednisolone pulse therapy were administered again on days 5–7. A subsequent direct antigen test of a bullae sample was positive for HSV-2, and a polymerase chain reaction (PCR) assay of the peripheral blood mononuclear cells (PBSCs) detected HSV-2 DNA. Moreover, tonsillar and esophageal biopsies revealed viral inclusion bodies. Immunohistochemical staining and quantitative real-time PCR from the formalin-fixed paraffin-embedded tissues confirmed the presence of HSV-2 but not HSV-1 (Fig. 3). The methods for immunohistochemical staining [5] and quantitative real-time PCR to test for HSV-1, − 2 [6], and β-actin [7] were described previously. Based on these results, disseminated HSV-2 infection with multiple bullae, tonsillitis, and esophagitis was diagnosed. Hepatic involvement was also suspected. It is worth noting that the patient denied any past history of oral or genital HSV infection. A lumbar puncture was not performed due to the lack of neurological deficits or symptoms.

The patient improved steadily on a two-week course of intravenous acyclovir. His hematological status and liver function normalized, and his cutaneous skin lesions resolved. He was discharged on day 22 in good general health and continued to receive oral valacyclovir for viral suppression due to his immunosuppressed status. Prednisolone was tapered gradually to 12.5 mg on discharge. No recurrence has been observed 7 months after the treatment.

## Discussion and conclusions

In the present case, pharyngotonsillitis developed as an initial manifestation of disseminated HSV-2 infection without genital involvement. To the best of our knowledge, no previous case with such a clinical course has been reported. In general, viral causes of tonsillitis include rhinovirus, respiratory syncytial virus, Epstein-Barr virus, parainfluenza virus, influenza virus, coxsackievirus, adenovirus, etc [8] The majority of herpetic pharyngotonsillitis cases in adults are due to HSV-1, but a few reports have shown that HSV-2 can also produce the same symptoms in sexually active patients [9, 10]. However, the present patient denied any oral sexual activity during a period of several months and had no sign of genital involvement.

In our search of the PubMed database (http://www.ncbi.nlm.nih.gov/pubmed) using the search terms, “disseminated,” “herpes simplex virus,” and “type 2,” we identified 15 case reports of adult disseminated HSV-2 infection in the English-language literature since 2000 [11–21]. Case reports not published in English, pediatric cases (patients younger than 15 years old), and review articles were excluded. We defined disseminated HSV-2 infection as any case with positive HSV-2 IgM antibody test results, the presence of HSV-2 detected by DNA-PCR in the PBSCs, or the concurrent involvement of two or more noncontiguous sites. Our case and 15 other reported cases are summarized in Table 1. The patients’ mean age was 28 (range: 18–67) years. Eight patients (50.0%) were male and eight (50.0%) were female. Six patients (37.5%) were treated with immunosuppressive agents at the onset of HSV-2 infection. Regarding the site of infection, 11 patients (68.8%) presented with liver involvement and six (37.5%) presented with a skin infection. Interestingly, including the present case, genital symptoms were not observed in nine patients (56.3%). The patients denied any past history of sexually transmitted infection. The etiology of the HSV-2 infection was obscure in most cases. Fourteen patients (87.5%) received intravenous acyclovir and ten (71.4%) responded to treatment.

**Table 1:** Adult disseminated HSV-2 infection: 15 reported cases and the presented case

Year, author [reference]	Age (y)/Sex	Underlying condition or disease	Immunosuppressive therapy at the onset	Infected organs	Complications	Treatment	Outcome
2005, Yamaguchi K et al. [11]	N/A / F	Pregnancy	None	Genital, skin	HLH	ACV	Survived
2006, Czartoski T et al. [12]	28 / F	Immunocompetent	None	Liver, skin	Candidemia	ACV	Died
2006, Ramasamy K et al. [13]	33 / M	Bone marrow failure of unknown etiology	Oxymetholone, prednisolone	Skin	HLH, rhabdomyolysis	ACV	Died
2007, Abbo L et al. [14]	51 / M	Immunocompetent	None	Liver, colon	FHF	ACV	Died
2010, Peppercorn A et al. [15]	58 / M	Burn	None	Skin, liver, lung	Septic shock	BSC	Dead
2012, Lee L et al. [16]	24 / F	AIDS	None	Pharynx, genital	None	ACV	Survived
2015, Rimawi BH et al. [17]	25 / F	Immunocompetent	None	Liver, skin, genital	None	ACV	Survived
2015, Rimawi BH et al. [17]	24 / F	Immunocompetent	None	Liver, skin	None	ACV	Survived
2015, Rimawi BH et al. [17]	27 / F	Pregnancy	None	Liver, genital	None	ACV	Survived
2015, Erdmann N et al. [18]	22 / M	Atopic dermatitis	Systemic corticosteroids	Skin, genital	None	ACV	Survived
2016, Down C et al. [19]	67 / M	Prostate cancer	None	Liver, genital	Ireus	ACV	Survived
2016, Ikumi K et al. [20]	56 / M	Muscle sclerosis	Methylprednisolone pulse therapy	Liver, rectum, adrenal grand etc.	HLH	None	Died
2017, Natu A et al. [21]	23 / M	Crohn’s disease	Vedolizumab, prednisolone	Liver, mouth, genital	Crohn’s flare	ACV	Survived
2017, Natu A et al. [21]	18 / F	Pregnancy	None	Liver	Septic shock, FHF	ACV	Died
2017, Natu A et al. [21]	65 / F	Liver cirrhosis	Liver transplantation	Liver	None	ACV	Survived
Presented case	46 / M	Hypereosinophilic syndrome	Azathioprine, prednisolone	Tonsil, esophagus,skin	HLH	ACV	Survived

*Abbreviations*: *ACV* acyclovir, *AIDS* acquired immune deficiency syndrome, *BSC* best supportive care, *M* male, *F* female, *FHF* fulminant hepatic failure, *HLH* hemophagocytic lymphohistiocytosis, *y* year

One plausible explanation of the pathogenesis of the present case is a reactivation of HSV-2 infection. Tobian et al. reported a case in which the initiation of antiretroviral therapy (ART) for HIV infection reactivated HSV-2, possibly via immune reconstitution inflammatory syndrome (IRIS) [22]. IRIS describes a collection of inflammatory disorders associated with an apparently paradoxical worsening of preexisting infectious processes following the initiation of ART in HIV-positive individuals [23]. Similar illnesses also occur in some HIV-negative patients after tapering immunosuppressive drugs [24–26]. In our case, we didn’t assess serological data using pre-serum specimens to confirm the past infection of HSV-2. However, the prednisolone dosage was gradually tapered due to the favorable clinical course of the hypereosinophilic syndrome, which suggests the possibility of HSV-2 reactivation accompanied by IRIS-like phenomenon. Physicians should consider the possibility of viral reactivation in patients receiving immunosuppressive agents if they encounter an infection of unknown etiology.

In the present case, the disseminated HSV-2 infection triggered the development of HLH. Ramos-Casals M et al. reported that *Herpesviridae* account for 62% of cases of virus-associated HLH including Epstein-Barr virus (43%), cytomegalovirus (9%), and other herpes viruses (10%) [27]. There are very few reports of HLH caused by HSV-2 [11, 13, 20]. Thus, no optimal treatment strategy for HLH caused by HSV-2 has yet been established. Several previous studies reported that patients recovered from infection-associated HLH through the specific treatment of the underlying infection and supportive care alone [28]. On the other hand, when an HSV-2 infection is disseminated, HLH can progress to multiple organ failure with an extremely poor prognosis [13, 20]. Our patient was treated successfully with intravenous acyclovir and methylprednisolone pulse therapy due to early diagnosis. The treatment of HLH might require an individual approach depending on the underlying trigger, disease severity, and clinical course.

The present study reported the first case of disseminated HSV-2 infection associated with pharyngotonsilitis, esophagitis, and HLH without genital involvement in an immunocompromised host. The patient recovered after receiving intravenous acyclovir and methylprednisolone pulse therapy. HSV-2 pharyngotonsillitis is rare and difficult to diagnose, especially if genital lesions are not present. This report emphasizes and promotes awareness of the importance of the early recognition and appropriate clinical management of HSV-2 infection. Additional data from similar cases and further studies to characterize HSV-2 infection and HLH in adults, particularly those who are immunocompromised, are needed.

## Abbreviations

ALT: Alanine aminotransferase
ART: Antiretroviral therapy
AST: Aspartate aminotransferase
HIV: Human immunodeficiency virus
HLH: Hemophagocytic lymphohistiocytosis
HSV: Herpes simplex virus
IgM: Immunoglobulin M
IRIS: Immune reconstitution inflammatory syndrome
LDH: Lactate dehydrogenase
PBSCs: Peripheral blood mononuclear cells
PCR: Polymerase chain reaction
